# Suppression of Akt-mTOR Pathway-A Novel Component of Oncogene Induced DNA Damage Response Barrier in Breast Tumorigenesis

**DOI:** 10.1371/journal.pone.0097076

**Published:** 2014-05-08

**Authors:** Anjana Bhardwaj, Daniel Rosen, Mei Liu, Yan Liu, Qiang Hao, Nivetha Ganesan, Carol J. Etzel, Ashley Gullett, Constance T. Albarracin, Isabelle Bedrosian

**Affiliations:** 1 Department of Surgical Oncology, The University of Texas M. D. Anderson Cancer Center, Houston, Texas, United States of America; 2 Department of Epidemiology, The University of Texas M. D. Anderson Cancer Center, Houston, Texas, United States of America; 3 Department of Pathology, The University of Texas M. D. Anderson Cancer Center, Houston, Texas, United States of America; University of Texas Health Science Center at San Antonio, United States of America

## Abstract

DNA damage has been thought to be directly associated with the neoplastic progression by enabling mutations in tumor suppressor genes and activating/and amplifying oncogenes ultimately resulting in genomic instability. DNA damage causes activation of the DNA damage response (DDR) that is an important cellular mechanism for maintaining genomic integrity in the face of genotoxic stress. While the cellular response to genotoxic stress has been extensively studied in cancer models, less is known about the cellular response to oncogenic stress in the premalignant context. In the present study, by using breast tissues samples from women at different risk levels for invasive breast cancer (normal, proliferative breast disease and ductal carcinoma in situ) we found that DNA damage is inversely correlated with risk of invasive breast cancer. Similarly, in MCF10A based in vitro model system where we recapitulated high DNA damage conditions as seen in patient samples by stably cloning in cyclin E, we found that high levels of oncogene induced DNA damage, by triggering inhibition of a major proliferative pathway (AKT), inhibits cell growth and causes cells to die through autophagy. These data suggest that AKT-mTOR pathway is a novel component of oncogene induced DNA damage response in immortalized ‘normal-like’ breast cells and its suppression may contribute to growth arrest and arrest of the breast tumorigenesis.

## Introduction

The process of tumorigenesis involves several genotoxic insults such as mutations, replicative stress and genetic instability [1]–[3]. These genotoxic insults lead to activation of DDR, a cellular mechanism for maintaining genomic integrity in the face of genotoxic stress- an important barrier against early stages of human tumorigenesis, leading to cell-cycle blockade or apoptosis and thereby constraining tumor progression. Known mechanisms of DDR barrier activated by oncogenes involves deregulating entry into the cell cycle directly or indirectly by enhancing the activities of CDKs that have a role in the G1 and S phase of cell cycle [4]. These deregulations in cell cycle often leads to unscheduled DNA replication that causes DNA damage or replicative stress. DNA damage is sensed by MRN (MR11-RAD50-NBS1) complex that leads to phosphorylation and activation of ATM and ATR kinases that subsequently cause the stabilization and activation of p53 [5]. Whereas oncogene induced aberrant replication evokes activation of ATR and chk1 both of these mechanisms together lead to the accumulation of phosphorylated form of γH2AX, a well recognized marker of DNA damage. In turn this results in cell cycle arrest or cell death in p53 dependent manner, creating a selection pressure against early tumor progression by removing the aberrant cells [5]. Various components of DDR barrier such as γH2AX, pChk2, p53 accumulation, focal staining of p53 binding protein 1 (53BP1) and apoptosis have been found to be activated in precancerous lesions from organs such a lung, colon, and skin suggesting the protective role of DDR in the process of neoplastic transformation [3]. Interestingly, these studies have reported tissue specific differences in the levels of proliferation and cell death/senescence in various stages of neoplastic transformation [3]. We were interested to study various components of DDR in mammary precursor lesions. The process of mammary tumorigenesis involves several well-characterized intermediate lesions. These lesions, such as hyperplasia and atypia, are associated with increased risk of invasive breast cancer; however, many women with these early histologic changes do not progress to carcinoma. The mechanisms that promote or inhibit progression of these intermediate breast lesions are not known. We sought to examine the role of DDR barrier in the progression of breast tumorigenesis by examining biomarkers of DNA damage, DDR activation, and cellular response in the breast tissues from women at different risk for development of invasive breast cancer. Furthermore, since DDR studies to date have been largely confined to tumor models, an additional objective of this study was to examine the mechanisms behind DDR activation in a non-tumor context, specifically looking at immortalized mammary epithelial cells.

## Materials and Methods

### Ethics Statement

Approval for this study was obtained from the institutional review board of the University of Texas, MD Anderson Cancer Center that also provided waiver of consent for performing and publishing these analyses.

### Patient Samples

Archived paraffin embedded tissue blocks from 3 cohorts of women were selected at random for the creation of tissue microarrays. The selection criteria were as follows: 1. Group A, women with histologically normal findings at time of breast biopsy and no personal history of breast cancer, 2. Group B, women with histologic findings of risk [atypical ductal hyperplasia (ADH), atypical lobular hyperplasia (ALH), lobular carcinoma in situ (LCIS)] but no personal history of breast cancer, 3. Group C, women with ductal carcinoma in situ (DCIS). For each case, up to 5, 1 mm cores were transferred to a TMA block. After processing, unstained slides from the TMA block were submitted for immunostaining of the biomarkers of interest as detailed below.

### Cell lines

MCF10A breast cancer progression model, comprising of MCF10A, MCF10A neoT, MCF10AT, MCF10A DCIS, MCF10A cA1d, and MCF10A cA1h, a series of already developed and published [6]–[9] cell lines originated from human breast epithelial cells MCF10A was used in the study. MCF10A, the ‘normal-like’ immortalized mammary epithelial cell lines (has lost p16) was obtained from ATCC and the other cell lines of the MCF10A model were obtained from the Karmanos Cancer Institute, MI. These cell lines share same genetic background, and represent various sequential advanced stages of breast cancer. All these cell lines were STAR tested by ATCC and Karmanos, and all the experiments using these cell lines were performed with in first 15 passages.

### Immunostaining

Immuno-staining was performed for DDR biomarkers, γH2AX, pP53 (on ser15), caspase-3 and Ki-67 by using automated “Autostainer 360 Lab Vision, Fremont, CA, USA” according to the manufacturer's protocol. Briefly, the sections were first deparaffinized, rehydrated and then antigen was retrieved. The non specific signal was blocked by incubating with hydrogen peroxide for 30 minutes, followed by washing with PBST followed by blocking with ultra V block for 5 minutes to block nonspecific binding. The tissue sections were then incubated with primary antibody (1∶500 dilution for γH2AX, 1∶100 dilution for pP53 and 1∶300 dilution for caspase-3, Ki-67) for 1 hr, washed once with PBS followed by incubation with primary antibody enhancer for 10 minutes. After washing with PBS, the sections were incubated with HRP polymer for 15 minutes, and the color was developed by adding DAB plus chromogen and substrate for 5 minutes and the counterstaining was performed with hematoxylin by staining for 1 minute. We also ran negative controls of non-immune serum in order to check the specificity of the immunostaining. Percent of cells stained were quantified using the Aperio automated imaging system as explained elsewhere [10].

### Generation of cyclin E expressing stable breast cell clones

To study the effect of oncogene over expression in normal mammary epithelial cells, we developed a model system of constitutively over expressing cyclin E by stably transfecting CMV promoter driven-FLAG-tagged cyclin E in MCF10A (P), human mammary epithelial cells. MCF10 (P) is already published, well established, one of the very few immortalized ‘normal like’ (has lost p16) mammary cell lines developed [6]–[9]. The full length cyclin E cloned in pcDNA 3.1 backbone (as described elsewhere [11]) was transfected into the MCF10A (P) cells by using Fugene 6 (Roche Diagnostics). After transfection, the cells were placed in medium containing 100 µg/ml G418 (Invitrogen), and individual colonies were isolated and propagated. We identified positive clones by assessing the expression of cyclin E by Western blot analysis using anti-cyclin E antibody. We also assessed the expression of transfected Flag-Cyclin E mRNA by QPCR using forward primer in the 3′ end of coding sequence of cyclin E and a reverse primer in the flag tag. For our present studies we selected two stable clones (named CyE C3 and CyE B10) possessing different expression levels of cyclin E. CyE B10 expressed more than 10-fold cyclin E transcript levels as compared to CyE C3 that showed more than 100- folds expression as compared to MCF10A (P) cells (“Figure S1a in File S1”), and the cyclin E protein also showed a similar trend in cyclin E expressing clones (“Figure S1b in File S1”).

### Analysis of γ-H2AX foci formation by Immuno-fluorescence followed by confocal microscopy

Cells were grown on cover slips in 6 well dishes for 24–48 h till they reach to about 60-70% confluency. The cells were washed with PBS and then fixed and permeabilized with chilled methanol for 15 min. The fixed cells were then washed with PBS, and blocked with 5% BSA in PBS for 1 h and incubated overnight with γH2AX primary antibody (1∶500 dilution in 1%BSA/PBS; Upstate, Charlottesville, VA, USA). The following day cells were washed with PBS, incubated with AlexaFluor 594-conjugated goat anti-rabbit secondary antibody (1∶200 dilution; Molecular Probes, Eugene, OR) for 1 h, washed and mounted with ProLong Gold antifade reagent with DAPI (Molecular Probes). Slides were imaged with a Olympus confocal microscope- IX-81 DSU from Olympus, Inc., Thornwood, NY, USA) using a 100× objective lens. Images of representative cell populations were captured, and γH2AX foci were counted visually. At least 100 cells were counted per cell clone, and each experiment was performed three times.

### Western blotting

We studied Akt-mTOR pathway in our model systems by measuring several molecules of the pathway including, AKT, pAKT, mTOR, pmTOR, FOXO1, pFOXO1, pS6, S6, p-4EBP-1 using Odyssey Infrared Imaging System. Briefly, 30–40 µg of total cell lysates were electrophoresed in SDS–polyacrylamide, transferred to Hybond ECL nitrocellulose (Amersham), and probed with the aforementioned antibodies or the loading control, vinculin. A single western membrane was probed for more than two proteins of interest (of different sizes by cutting the membrane) and a loading control, vinculin because of the ability of Odyssey Infrared Imaging System to detect signals from antibody raised in mouse and rabbit to be used on same membrane and measured at separate wavelengths. As a result, in our western blot figures one common vinculin protein band is shown for multiple proteins if these all came from same membrane.

### Growth curve assays

Cells were plated in 24- well plates at a density of 10,000 cells/well. Media was replaced every other day until day 20 post plating. Cell counts were determined every other day by trypsinizing the cells and replating. The cell number was plotted against days for each cell line, and the doubling time was calculated by using an online tool (http://www.doublingtime.com/compute. php, [12].

### Autophagy

Autophagy was determined in MCF10A cells and cyclin E over-expressing MCF10A clone (2 × 10^5^ cells) by staining cells with 1 µg/ml acridine orange for 15 minutes at 37°C in the dark and sorting the dead cells stained by flow cytometry. Upon staining with acridine orange, the cytoplasm and nucleolus fluoresce bright green and dim red, whereas acidic compartments, representing cell death through autophagy, fluoresce bright red. After trypsinizing the cells, green (510–530 nm) and red (650 nm) fluorescence emission from illumination with blue (488 nm) excitation light was measured on FACSCalibur (Becton Dickinson) and quantified using CellQuest software. MCF-10A cells treated with chloroquine were used as a positive control for autophagy.

### Statistical analysis

Statistical analyses for assessing risk level for invasive breast cancer by using DNA damage associated cellular markers were performed using the SAS statistical software package (SAS Institution Inc., Cary, NC) and S-Plus software (Insightful Corporation, Seattle, WA). All *P* values are two-sided, and *P* values less than or equal to 0.05 were considered statistically significant. Each biomarker was analyzed as both a continuous and a categorical (positive vs. negative) variable. Fisher's exact test was used to test for the difference of the biomarker positive rate between the risk groups. Patient samples were considered evaluable if at least one core contained ductal epithelium. Since each patient could therefore have a different number of cores included for each biomarker analysis, the mean difference among the three risk cohorts was compared using a general linear model with the adjustment of number of cores evaluated. We also used classification and regression tree (CART) analysis to identify possible distinct combination of the biomarkers that are associated with the different risk level for breast cancer. We built a decision tree to discriminate risk groups using recursive partitioning technique “rpart” package that was developed for S-Plus. We grew the decision tree with the stipulation that each subsequent split yields two daughter notes with at least 10 participants per node. For all other assays analysis of variance (ANOVA) followed by Dunnetts' test was performed. *P* values less than or equal to 0.05 were considered statistically significant.

## Results

### High levels of DNA damage and low levels of proliferation are associated with low risk for breast cancer

In order to address if DNA damage associated cellular markers possess any correlation with the risk for invasive breast cancer, we investigated 3 cohorts of women: i) women with histologically normal breast tissue (group A, n = 50), ii) women with histological changes of increased risk (LCIS, ADH, ALH) (group B, n = 54) but without a personal history of breast cancer and iii) women with DCIS (group C, n = 46) (Table 1). We studied cell proliferation (ki-67), apoptosis (caspase 3), DNA damage (γH2AX) and ser-15 p53 in breast tissue samples from these women. Similar to previous reports [13], higher levels of ki-67 were significantly associated with increasing risk for invasive breast cancer (p<0.001) (Table 1). However, surprisingly, we found that the proportion of cells staining for γH2AX significantly decreased (p<0.001) with increasing risk lesions (Table 1, “Table S1 in File S1”), indicating that DNA damage is inversely correlated with invasive breast cancer risk. Caspase-3 and ser15-p53 levels were not significantly different between the 3 risk groups (all p>0.05). Given the limited representation of tissue from each case, we tested for heterogeneity between cores from each case for each biomarker to ensure that the findings were not biased by sampling errors. The results for all 4 biomarkers suggested that variance within a patient is limited (heterogeneity [*e*] <0.5), thus minimizing the effects of staining heterogeneity on the results presented (Table 1, “Table S2 in File S1”).

**Table 1:** Frequency (% staining) of biomarker expression in cohorts with different risk levels for invasive breast cancer.

	DNA Damage γH2AX (%)	Proliferation Ki-67	Apoptosis Caspase-3 (%)	DDR sensor p-p53(%)
**Group A (Average risk) N = 50**	79.7+2.1	1.3+1.5	1.9+0.3	14.4+2.0
**Group B (ADH/ALH/LCIS) N = 54**	76.8+2.0	4.1+1.4	1.7+0.2	19.1+1.8
**Group C (DCIS) N = 46**	69.8+2.1	11.4+1.6	1.9+0.3	18.0+2.1
**p (Group A vs. C)**	0.001	0.001	0.869	0.243

Values are represented as mean + SE.

**Figure 1:**
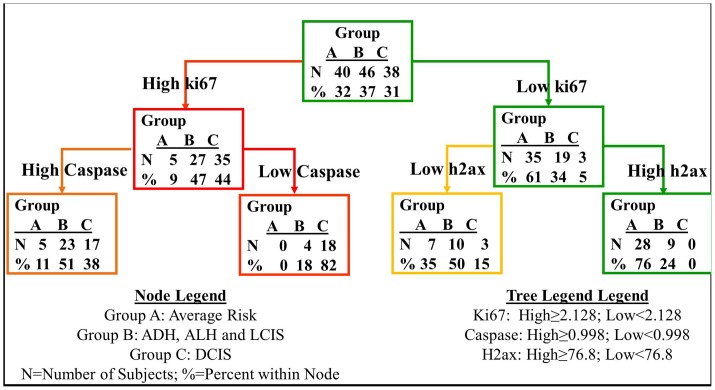
DNA damage in breast cancer risk and progression (a) Tree modeling showing predictability power of combination of several biomarkers for invasive breast cancer risk. Note that starting node in the tree model is fewer number than the total given for each risk cohort (40, 46 and 38). This reflects cases that were removed because all three biomarkers- Ki67, γ-H2AX and Caspase-3 -were not evaluable. Although this resulted in a smaller subset, this was necessary to better identify the distinct combination of the biomarkers by growing the tree based on completed data for all biomarkers of interest.

Giving our surprising finding that DNA damage was inversely associated with risk, we further explored the relative hierarchy of our biomarkers in predicting for breast cancer risk to better understand the potential role that DNA damage may play in breast tumorigenesis. We performed tree modeling to identify cut points and association of the various biomarker combinations with invasive breast cancer risk (Fig. 1a). In order to test the feasibility of creating a tree model, we first tested the statistical power of the number of samples used in the study to make sure that our sample size is large enough to draw any conclusions. Specifically, we used the PASS program (http://www.ncss.com) to calculate minimal effect size among the three risk groups. With 80% power and a conservative alpha level of 0.01(to account for multiple comparisons), we would be able to detect an effect size as small as 0.30 with our group sizes of 50, 54, and 46. From our preliminary results for the four biomarkers, we observed effect size as small as 0.78 (Caspase-3) and as large as 6.5 (Ki-67), therefore, suggesting that our selected sample size had sufficient power for the planned analysis. Computer generated cut-points were created for ki-67, γH2AX and caspase-3 at 2.128, 76.8 and 0.998 respectively. We also included p-p53 in the tree model initially; however, based on the stipulation to grow the decision tree that each subsequent split should yield two daughter nodes with at least 10 subjects per node, we removed p-p53, because it was the split node with less than 10 subjects for each daughter node. Although caspase-3 was not significant in the individual biomarker analysis, it was nonetheless of clinical interest and thus included in the multi-marker model. As shown in Fig.1a, this model identified that the *combination of high* γ*H2AX and low proliferation was disproportionately associated with women at average risk for breast cancer*. Seventy-six percent of average risk group (group A) fell into this combination compared with 24% of those with histologic changes of increase risk (group B) and 0% of those with DCIS (group C). Further analysis of the tree model to determine the relative contribution of each of these 3 biomarkers to the cancer risk placed ki-67 as the highest node within this hierarchy. However, high levels of γH2AX added additional specificity to the model and allowed for the identification of a clear average (low) risk state. This analysis suggested that one potential cellular barrier to DNA damage was arrest of cell proliferation and we next sought to identify potential pathways that may be involved in mediating arrest of cell growth following oncogene induced DNA damage.

### Cyclin E induced DNA damage alters growth characteristics in MCF10A cells

**Figure 2:**
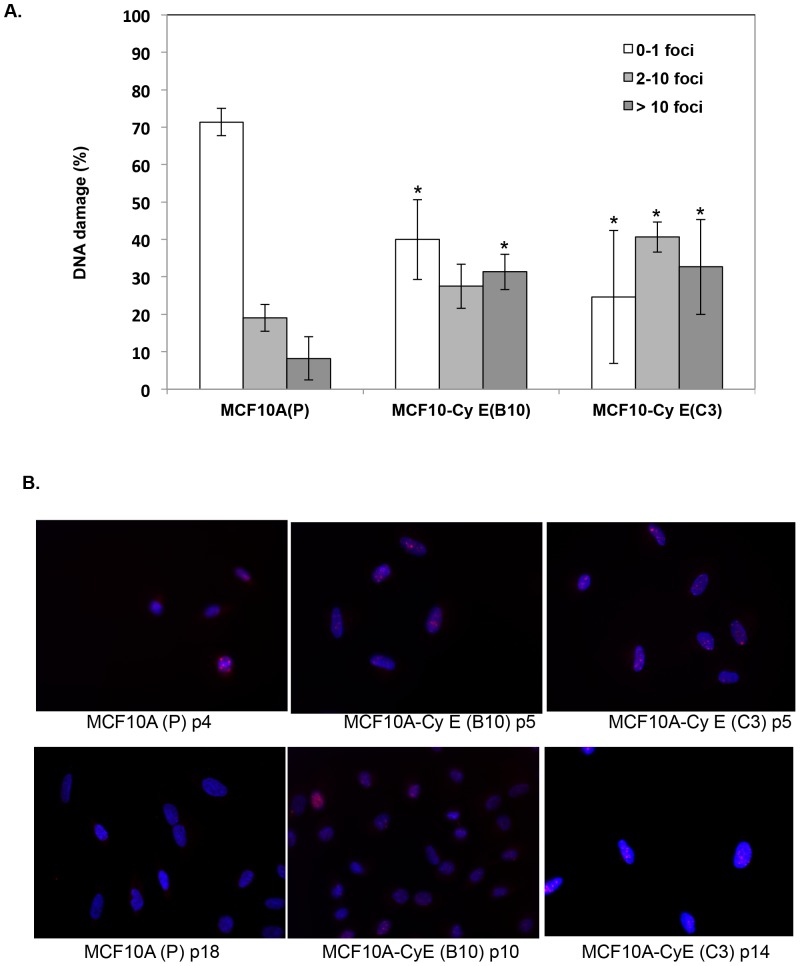
Cyclin E over expression increases DNA damage in normal breast cells. **(a)** Bar diagram shows the expression level of γ H2AX, a marker of DNA double strand break, in terms of number of foci per cell as measured by immunofluorescence (IF) in MCF10A parental (P) or cyclin E over expressing cell clones {MCF10A CyE (B10) and MCF10A CyE (C3). The cells with 0–1 foci were considered with no DNA damage, 2–10 foci, were considered as moderate DNA damage and > 10 foci was considered as high levels of DNA damage. The values shown are mean + standard error obtained by counting more than 100 cells and was repeated 3 times. An asterisk (*) indicates the statistically significant differences from MCF10A (P) at p < 0.05. **(b)** Photomicrographs at 100× showing the nuclear accumulation of γH2AX by using IF technique where alexa-594 conjugated secondary anti-rabbit antibody was used to stain the γH2AX as red color and DAPI was used as a counter stain to visualize nuclei.

**Figure 3:**
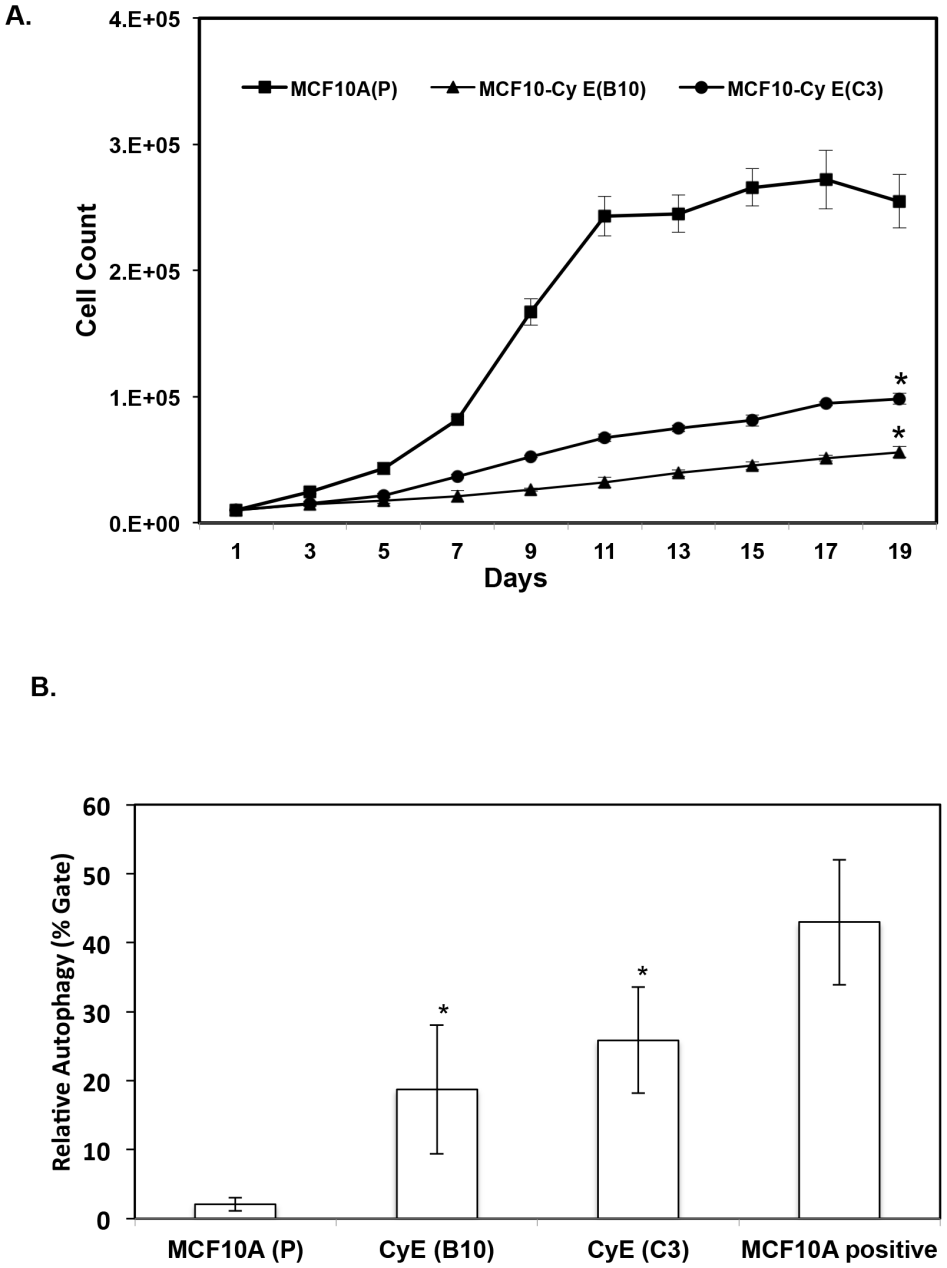
Cyclin E over expression inhibits cell growth through autophagy. **(a)** line diagram shows the growth curve of MCF10A parental (P) or cyclin E over expressing cell clones {MCF10A CyE (B10) and MCF10A CyE (C3). 10,000 cells were plated in 24-well plate and the growth pattern was studied by counting cells every other day by trypsinization followed by cell count using hemocytometer. The values shown are mean + standard error obtained by repeating the growth assay 3 times. An asterisk (*) indicates the statistically significant differences from MCF10A (P) at p < 0.05. **(b)** Bar diagram shows the % of cells dying through autophagy in cyclin E over expressing cell clones {MCF10A CyE (B10) and MCF10A CyE (C3) as compared to MCF10A parental (P). 100,000 cells were plated in 6-well plate and next day cells were stained by acridine orange, washed with PBS and sorted by flow cytometry. The values shown are mean + standard error obtained by repeating the cell cycle assay 3 times. An asterisk (*) indicates the statistically significant differences from MCF10A (P) at p < 0.05.

We developed an in vitro model of high DNA damage by overexpressing cyclin E that produced in our immortalized mammary epithelial cell line, MCF 10A cells. Cyclin E was chosen for these studies because cyclin E has been shown to be involved in breast tumorigensis [14] and cyclin E induced DNA damage has been mostly attributed due to DNA replication deregulation and independent of oxidative stress [15]. MCF 10A stable clones constitutively over expressing cyclin E were generated as detailed in the methods. Consistent with prior reports, cyclin E overexpression led to robust increase in the number of γH2AX foci as measured by IF (Fig. 2a,b). To determine how cyclin E induced DNA damage affects cell growth/proliferation, we performed a growth curve assay of parental MCF10A cells and the two cyclin E over expressing CyE (C3), and CyE (B10) cell clones. We found cyclin E clones to be slower growing than parental MCF10A cells with an increase in doubling time from 2.4 days in MCF10A (P) to almost 4 and 7 days in CyE (C3) and CyE (B10) respectively (p<0.05) (Fig. 3a).

### Cyclin E over expression causes mammary cells to die through autophagy

In order to understand if the decreased cell growth of CyE clone (C3) clone and CyE clone (B10) is due to cell death by apoptosis or autophagy, we studied both phenomenon in our cell clones. We found that although there was an increase in p53 levels in cyclin E expressing cell clones (data not shown) this did not correlate with a significant increase in apoptosis (“Figure S2 in File S1”). In contrast, we observed a robust increase (p<0.05) in the proportion of cells undergoing autophagy in both CyE clones (B10) (by almost 10- fold) and (C3) by more than 12- fold Fig. 3b).

### Cyclin E induced DNA damage leads to repression of AKT-mTOR signaling

**Figure 4:**
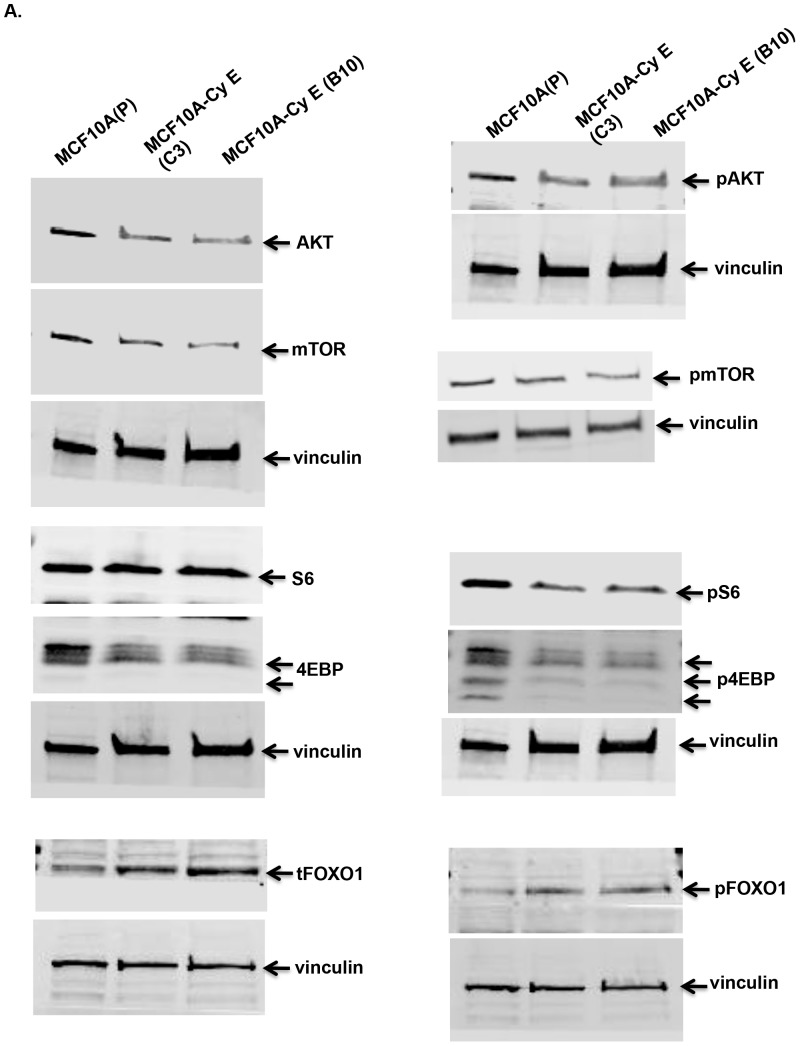
Cyclin E over expression suppresses AKT pathway. **(a)** Western blot analysis of the indicated proteins using whole cell lysates from MCF10A parental (P) or cyclin E over expressing cell clones {MCF10A CyE (B10) and MCF10A CyE (C3). Vinculin was used as loading control. Data is representative of three separate experiments.

To understand the mechanism behind the slower growth pattern and enhanced autophagy of cyclin E expressing cell clones we studied one of the key survival pathway, Akt-mTOR. Previously, computational studies have predicted several molecules of PI3 kinase pathway to be directly regulated by DNA damage [16]. Indeed, we found the Akt-mTOR pathway to be repressed in both CyE C3 and CyE B10 cell clones that possess increased DNA damage (Fig. 4). This repression was seen both at the level of inhibition in gene activity of several key players in Akt-mTOR signaling pathway as well as post-translational changes with reduced levels of some of the phospho-proteins within this pathway. Considering the fact that CyE C3 and CyE B10 cell clones are stable, the effects of cyclin E overexpression reflect results of long-term manipulations and hence are different from short-term treatments that usually lead to changes in post-translational modifications only [17]. Consistent with repression of Akt-mTOR signaling, we see an increase in expression of FOXO1. As a negative control we also tested the effects of empty vector (pcDNA 3.1) on AKT-mTOR pathway, and found that the empty vector backbone does not suppress AKT-mTOR pathway in transient transfections (“Figure S3 in File S1”) whereas cyclin E over expressing MCF10A cells did.

### Suppression of AKT-mTOR signaling is relieved by exposure to a second oncogenic stimulus/event

**Figure 5:**
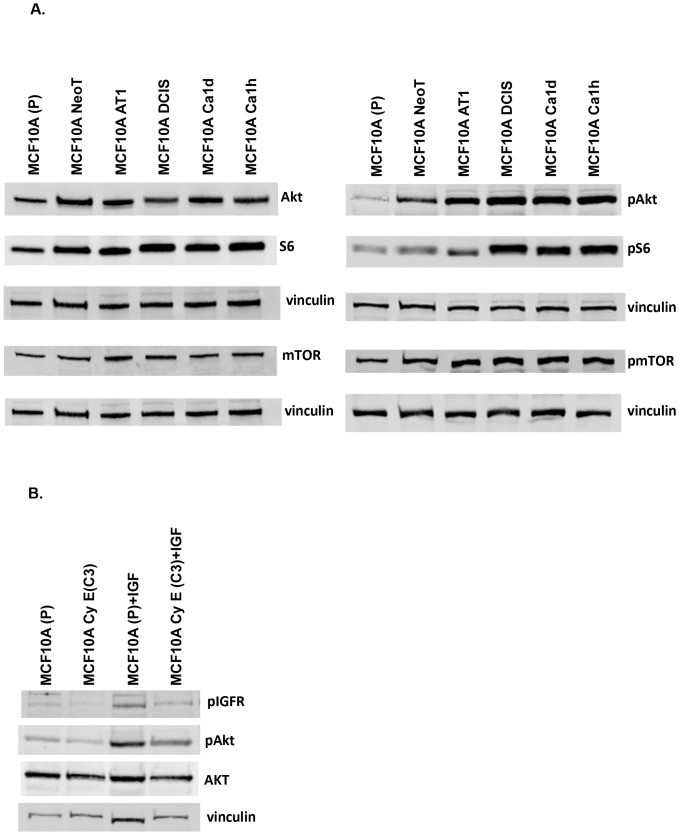
Activation of AKT–mTOR pathway during mammary tumorigenesis. **(a)** Western blot analysis of the indicated proteins using whole cell lysates from MCF10A parental (P) or MCF10A NeoT, MCF10AT1, MCF10.DCIS, MCF10 Ca1d, and MCF10 ca1h. Vinculin was used as loading control. Data is representative of three separate experiments. **b)** Western blot analysis of the indicated proteins using whole cell lysates from MCF10A parental (P) or cyclin E overexpressing cell clone MCF10A-cy E (C3) with and with out IGF1 treatment (25ng/ml for 20minutes). Vinculin was used as loading control. Data is representative of three separate experiments.

Our single oncogene model suggested that suppression of AKT-mTOR signaling might be a part of the barrier to tumorigenic progression. We further tested this phenomenon in the context of cancer progression model. Consistent with the hypothesis that AKT-mTOR may be part of the DDR barrier, we observed an activation of pAkt, and pS6 in the MCF10A progression model with levels increasing in MCF10.AT1, MCF10.DCIS and MCF10.CA1d as compared to MCF10A (P) [fig. 5a]. These data from the 2 model systems support the idea that the AKT-mTOR pathway may be involved in breast tumorigenesis and that suppression of this pathway may be part of the tumorigenic barrier. We were interested to see whether suppression of this growth pathway (in cyclin E transfected normal breast cells) can be overcome by exposure to a second oncogenic event, specifically IGF-1 that is known to activate this pathway. Figure 5b shows that exposure of cyclin E overexpressing MCF10A (C3) cells to IGF1 (25 ng/ml) relieves the suppression in AKT-mTOR pathway. These data are in line with the established paradigm of accumulation of multiple oncogenic events that lead to escape from DDR and progression towards an invasive cancer phenotype.

## Discussion

It is estimated that 75% of women who develop sporadic invasive breast cancer have no known epidemiological risk factors. Thus identification of tissue-based risk factors to identify early molecular changes within the histologically normal breast will provide a more precise and individualized assessment of breast cancer risk. In this report, we describe the presence of DNA damage foci in histologically normal breast tissue, in combination with low proliferation index, Ki-67, to be strongly associated with a low risk state. We also report that activation of DDR appears to be a robust barrier to mammary tumorigenesis, and that suppression of AKT-mTOR pathway may be involved in maintenance of this barrier. Activation of the DNA damage response (DDR) is an important cellular mechanism for maintaining genomic integrity in the face of genotoxic stress [18]. Multiple oncogenes have been shown to activate this checkpoint, suggesting a common DDR pathway that is up regulated largely independent of the original oncogenic stimulus [4]. The observation that mutations in DNA damage repair proteins: BRCA 1 and BRCA 2 in carriers families have been causally linked to the development of carcinoma [19] primarily in the breast and ovary suggest that DNA damage and repair are particularly relevant in the process of tumorigenesis in these organs. DNA DSB repair capacity has also been observed to be associated with increased susceptibility to cancer in sporadic cases as well. Single nucleotide polymorphisms of DNA repair genes have been associated with increased risk of breast cancer [20]–[24] and recently, variants of the ATM gene, a key regulator of the cellular response to repair DNA damage, has also shown to be associated with altered susceptibility towards breast cancer [25]. The relevance of DNA damage to breast tumorigenesis is also highlighted by the fact that breast tumors lose expression of MRN complex proteins (that maintain genomic integrity by sensing DNA damage and through repair) and these defects were even more pronounced in TNBC, where mutations in the genes responsible for DNA DSB repair (particularly NSB) have been linked to poor patient survival [26]. In a pioneering study, using invasive *breast cancer* samples, Bartikova *et al* very interestingly reported γH2AX positivity to be associated with TNBC stage and p53 aberration. In concordance with Bartikova et al [26], DCIS cases from the present study when subdivided in to groups based on hormone positivity revealed a trend of higher γH2AX staining in ER^−^/PR^−^ group (78%) as compared to the ER^+^ group (68%). These preliminary findings were not statistically significant (p = 0.27, ns) because of smaller sample size of ER^−^/PR^−^ group thus need to be explored further. These observations along with overall expression pattern of low γH2AX and high Ki67 in high cancer risk group is in concordance with Nagelkerke et al [27] where by using a cohort of lymph node negative patients (n = 122) the authors reported γH2AX to be positive in only about 8% cases (patient samples). Secondly, this study also reported γH2AX to be of prognostic value in TNBC, and BRCA1 [27]. Although, there are a couple of studies (as discussed herein) that have reported lower expression of proteins involved in DNA damage sensing and repair in invasive breast cancer but none of these have looked at these parameters in premalignant and higher risk lesions. Thus, *our study is the first to report on the tissue level assessment of DNA damage and breast cancer risk where we find that there is a significant inverse association, thus supporting the role of DDR as a tumorigenic barrier.*

Our data also suggests a novel mechanism that may be involved in maintaining the DDR barrier, namely the suppression of the AKT-mTOR pathway. Although the relevance of this pathway has been well documented in the setting of invasive breast cancer and metastasis, our findings suggest that Akt-mTOR signaling may also play a role in the setting of initiation and progression towards breast cancer in ‘normal-like’ breast cells. In the present study we find an *activation* of Akt-mTOR pathway during breast tumorigenesis, while in immortalized mammary epithelial cells subjected to oncogenic insult, there is significant induction of DNA damage and concomitant *suppression* of the Akt-mTOR pathway. This suggests that additional oncogenic insults may allow normal cells to bypass DDR and relieve the suppression of Akt-mTOR pathway. Indeed, we found this suppression of Akt-mTOR to be relieved by IGF1 treatment of MCF10A cyclin E clones. In addition previously studies have reported that gain of additional oncogenic mutations such as H-ras causes activation of Akt-mTOR pathway in the process of mammary tumorigenesis [28]–[30]. Studies of Young et al and Kim et al [28]–[30] showed that the more advanced stages of breast cancer over express several other oncogenic and signaling proteins such as IGF-1R, Cyclin D1, c myc, pERK, Stat3, and Pak4; some of which are known activators of Akt-mTOR pathway.

Although cell death subsequent to DNA damage has been widely reported to be through apoptosis, in this study we observed an increase in autophagy in cyclin E overexpressing cell clones. This is consistent with a recent report from Walker and coworkers on oxidative stress induced DNA damage leading to suppression in mTORC signaling and induction in autophagy [17]. In the study, Walker and coworkers showed that in response to oxidative stress, DNA damage sensor ATM through cytoplasmic function activates a tumor suppressor complex called tuberous sclerosis complex (TSC2) to repress mTORC1 in cytoplasm [17]. A similar cytoplasmic role of ATM may be at play in transducing oncogene induced DNA damage to suppress Akt-mTOR pathway as shown in the present study.

In summary, our findings suggest that the interplay between DNA damage and proliferation are key elements in normal mammary tissue that impacts the susceptibility of breast cells to transformation. Their interplay may in part be mediated through AKT-mTOR pathway; this information may be of clinical utility in improving our current risk stratification methods and providing new opportunities for targeting prevention.

## Supporting Information

File S1 **Supporting information figures and tables.** **Figure S1,** Cyclin E levels in MCF10A parental (P) or cyclin E over expressing stable cell clones {MCF10A CyE (B10) and MCF10A CyE (C3) **(a)** measured at RNA level by QPCR using primers that span cyclin E and Flag sequence thus show the levels of transfected flag tagged cyclin E in breast cells. **(b)** measured by immunofluorescence (IF) by using cyclin E antibody that picked up both the endogenous and transfected cyclin E levels. **Figure S2,** Cyclin E over expression does not cause cell death through apoptosis. **(a)** Bar diagram showing the % of cells undergoing early apoptosis, late apoptosis and necrosis in cyclin E over expressing cell clones {MCF10A CyE (B10) and MCF10A CyE (C3) as compared to MCF10A parental (P). 100,000 cells were plated in 6-well plate and next day cells were stained with Annexin V-FITC and PI and were sorted by flow cytometry. The values shown are mean + standard error obtained by repeating the cell cycle assay 3 times. **Figure S3,** Transient over expression of Cyclin E suppresses AKT-mTOR pathway in MCF10A parental (P) cells. **(a)** levels of indicated proteins were measured by western blotting. **Table S1,** P values for pair wise comparisons of the three groups. **Table S2,** The heterogeneity (*e*) and 95% CI of the biomarkers.
(PDF)
