# Supplementary material for: Suppression of Akt-mTOR Pathway-A Novel Component of Oncogene Induced DNA Damage Response Barrier in Breast Tumorigenesis
Source: PLoS One. 2014 May 8;9(5):e97076. doi: 10.1371/journal.pone.0097076 (PMC4014598; doi:10.1371/journal.pone.0097076)
Supplement: File S1 — Supporting information figures and tables. Figure S1, Cyclin E levels in MCF10A parental (P) or cyclin E over expressing stable cell clones {MCF10A CyE (B10) and MCF10A CyE (C3) (a) measured at RNA level by QPCR using primers that span cyclin E and Flag sequence thus show the levels of transfected flag tagged cyclin E in breast cells. (b) measured by immunofluorescence (IF) by using cyclin E antibody that picked up both the endogenous and transfected cyclin E levels. Figure S2, Cyclin E over expression does not cause cell death through apoptosis. (a) Bar diagram showing the % of cells undergoing early apoptosis, late apoptosis and necrosis in cyclin E over expressing cell clones {MCF10A CyE (B10) and MCF10A CyE (C3) as compared to MCF10A parental (P). 100,000 cells were plated in 6-well plate and next day cells were stained with Annexin V-FITC and PI and were sorted by flow cytometry. The values shown are mean + standard error obtained by repeating the cell cycle assay 3 times. Figure S3, Transient over expression of Cyclin E suppresses AKT-mTOR pathway in MCF10A parental (P) cells. (a) levels of indicated proteins were measured by western blotting. Table S1, P values for pair wise comparisons of the three groups. Table S2, The heterogeneity (e) and 95% CI of the biomarkers. (PDF) [file pone.0097076.s001.pdf]

**A.**

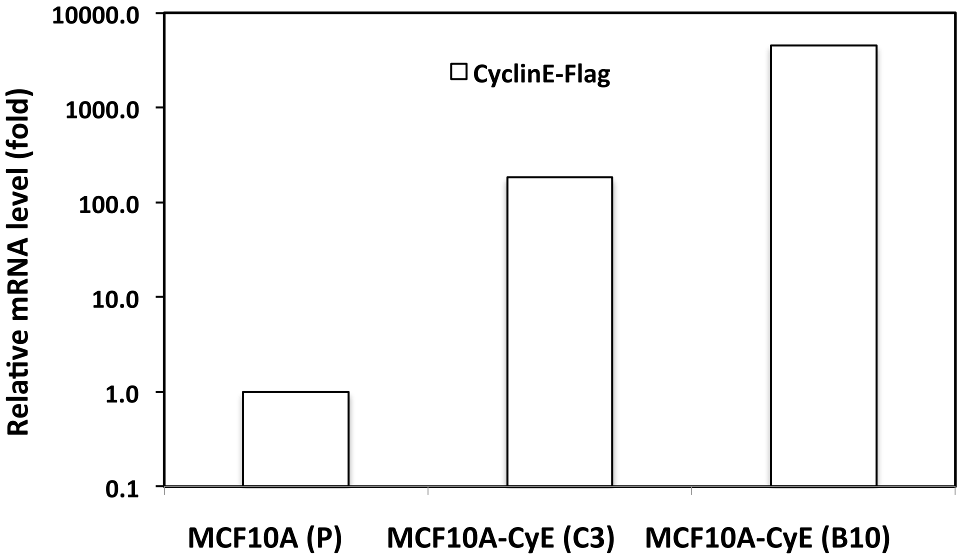


**Fig. S1** Cyclin E level in MCF10A parental (P) or cyclin E over expressing stable cell clones {MCF10A CyE (B10) and MCF10A CyE (C3) **(a)** measured at RNA level by QPCR using primers that span cyclin E and Flag sequence thus show the levels of transfected flag tagged cyclin E in breast cells. **(b)** measured by immunofluorescence (IF) by using cyclin E antibody that picked up both the endogenous and transfected cyclin E levels.

Fig. S1 (contd.)

B.

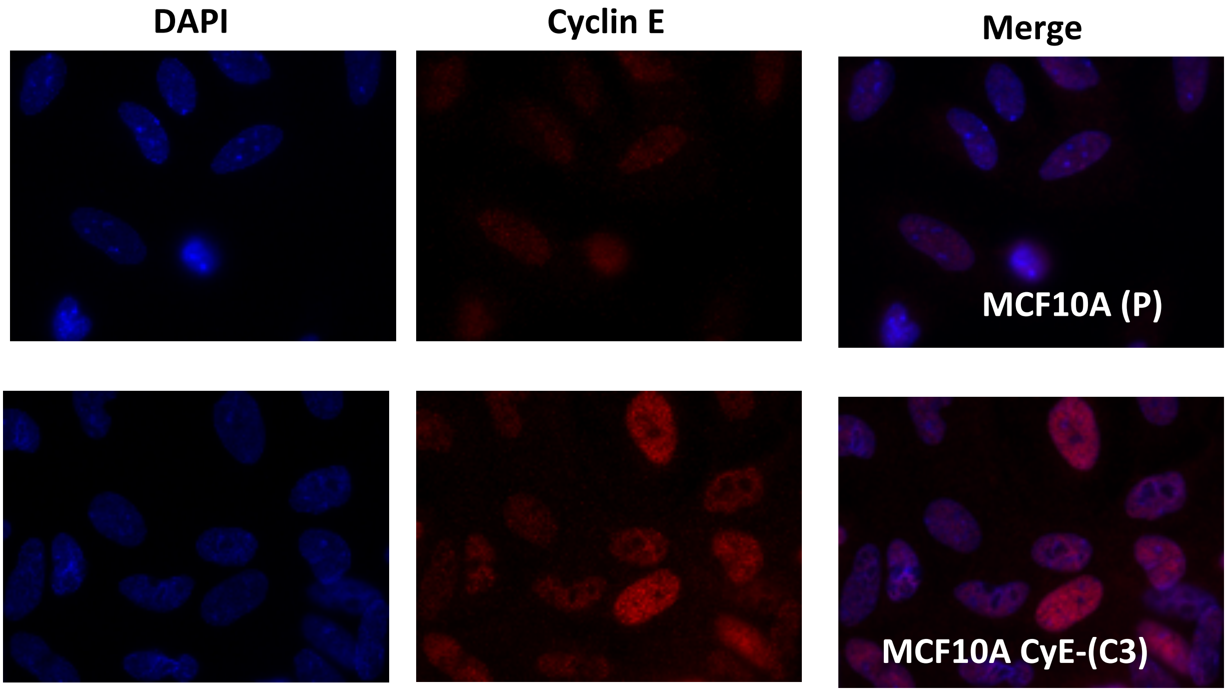


**Set I**
MCF 10A (P)
Vs.
MCF10A (C3)

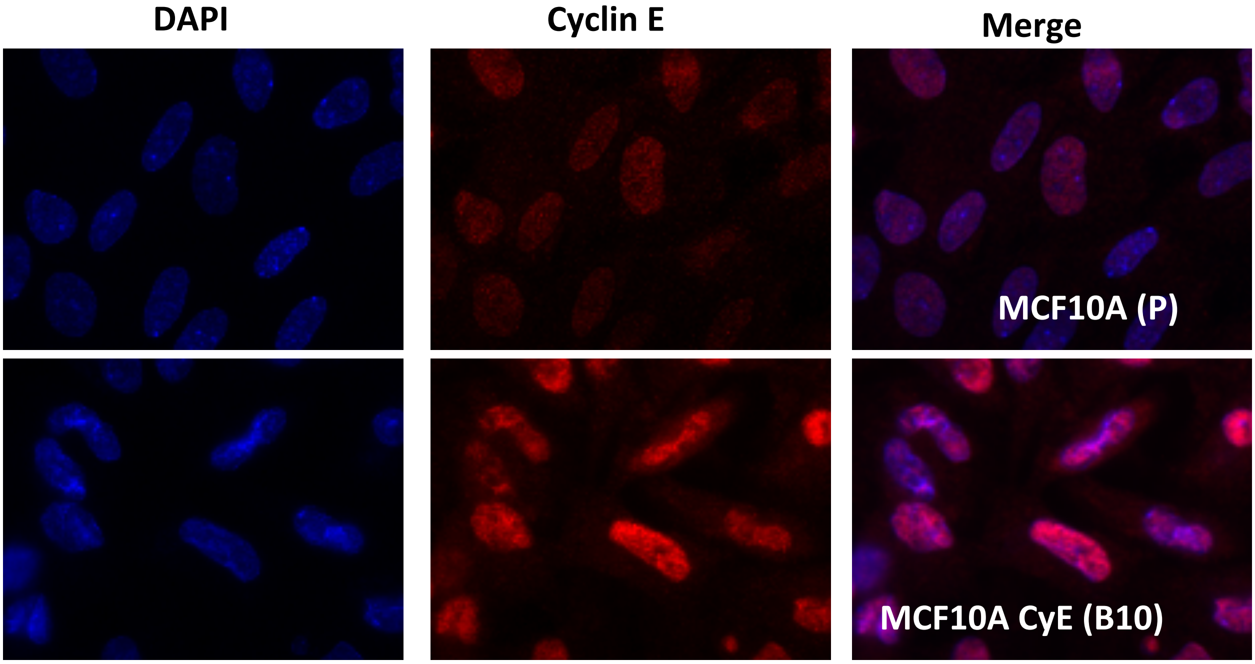


**Set II**
MCF 10A (P)
Vs.
MCF10A (B10)

**Fig. S2**

A.

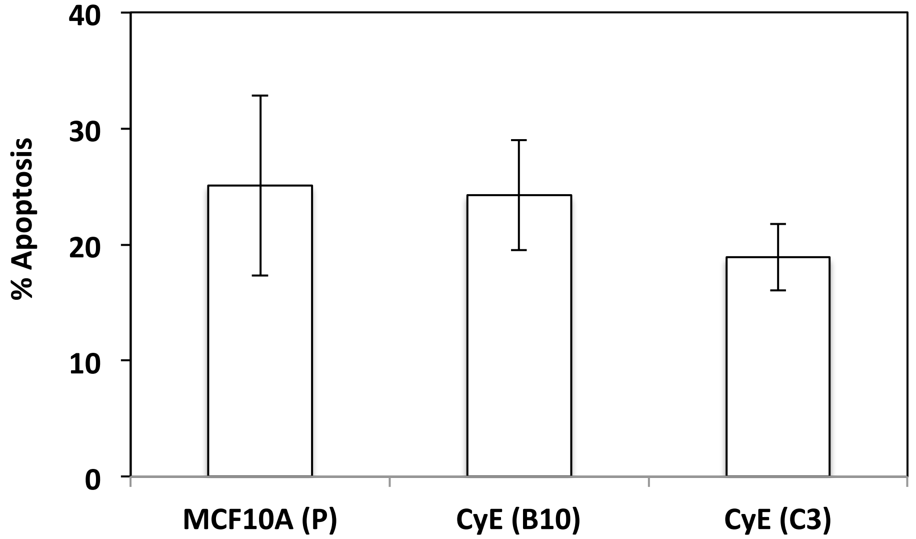


**Fig. S2** Cyclin E over expression does not cause cell death through apoptosis.
**(a)** Bar diagram showing the % of cells undergoing early apoptosis, late apoptosis and necrosis in cyclin E over expressing cell clones {MCF10A CyE (B10) and MCF10A CyE (C3) as compared to MCF10A parental (P). 100,000 cells were plated in 6-well plate and next day cells were stained with Annexin V-FITC and PI and were sorted by flow cytometry. The values shown are mean $\pm$ standard error obtained by repeating the cell cycle assay 3 times.

**Methodology: Apoptosis**

Apoptosis was determined in MCF10A cells and cyclin E overexpressing MCF10A clones by annexin V and propidium iodide (PI) staining by using FITC Annexin V Apoptosis Detection Kit II (BD Pharmingen). Briefly, the cells were harvested, washed with PBS and then resuspended in 1X annexin V binding buffer at a concentration of $1x10^6$ cells/ml. 0.1 ml of solution containing $1x10^5$ cells were incubated with 5µl of FITC Annexin V and 5µl PI (10ug/ml in PBS). Gently vortexed he cells and incubated for 15 min at RT in dark. Added 400µlof 1X annexin V binding buffer to each sample, analyzed by flow cyometry within 1hr and sorted the cells by FACS Calibur (Becton Dickinson) flow cytometry and quantified using Cell Quest software. Viable cells are both AnnexinV-FITC and PI negative. While cells that are in early apoptosis are AnnexinV-FITC positive and PI negative and cells that are in late apoptosis or already dead are both FITC Annexin V and PI positive.

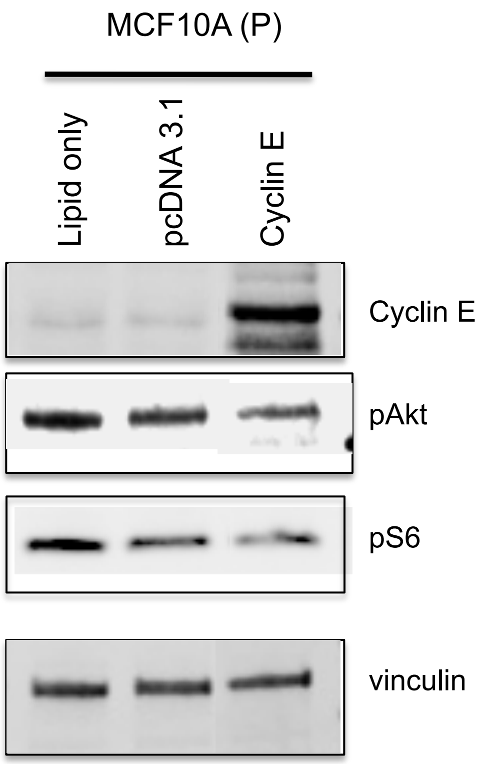


**Fig. S3** Transient over expression of Cyclin E suppresses AKT-mTOR pathway in MCF10A parental (P) cells. **(a)** levels of indicated proteins were measured by western blotting.

**Table S1**: P values for pair wise comparisons of the three groups

| Group Comparisons | DNA Damage γ-H2AX (%) | Proliferation ki-67 (%) | Apoptosis Caspase-3 (%) | DDR sensor p-p53(%) |
|---|---|---|---|---|
| **A vs B** | 0.321 | 0.154 | 0.692 | 0.077 |
| **A vs C** | 0.001 | 0.001 | 0.869 | 0.243 |
| **B vs C** | 0.018 | <0.0001 | 0.626 | 0.716 |

| **Table S2: The heterogeneity (*e*) and 95% CI of the biomarkers** | | | | |
|---|---|---|---|---|
| **Cohort** | **ϒ-H2AX** | **Ki-67** | **Caspase-3** | **Pp53** |
| **All** | 0.29(0.24, 0.35) | 0.20(0.16, 0.25) | 0.42(0.34, 0.50) | 0.32(0.26, 0.39) |
| **Group A (average risk)** | 0.29(0.20, 0.43) | 0.61(0.47, 0.73) | 0.45(0.32, 0.50) | 0.54(0.38, 0.71) |
| **Group B(ADH/ALH/ LCIS)** | 0.50(0.36, 0.63) | 0.22(0.15, 0.31) | 0.52(0.38, 0.67) | 0.36(0.26, 0.48) |
| **Group C(DCIS)** | 0.25(0.17, 0.33) | 0.15(0.10, 0.23) | 0.32(0.20, 0.52) | 0.14(0.08, 0.24) |

**Table S2** presents the heterogeneity (*e*) and 95% confidence intervals of each biomarker in cohorts, calculated as described by Pintilie et al. [1].
(Note that e = 0.5 means that the variance within different punches/ cores is equal to variance between patients. An *e* much larger than 0.5 indicates that the biomarker is highly variable within a patient and thus it can be anticipated that a representative value for a patient will be obtained with great difficulty.
The results shown in table 1 indicate the heterogeneity of the biomarkers for most cohorts is not so extensive, which means that there are not many erroneous measurements involved in the study.

[1] Pintilie M, Iakovlev V, Fyles A, Hedley D, Milosevic M, Hill RP, *Heterogeneity and power in clinical biomarker studies. J Clin Oncol 2009;27:1517–21*
